# AZD5363 Inhibits Inflammatory Synergy between Interleukin-17 and Insulin/Insulin-Like Growth Factor 1

**DOI:** 10.3389/fonc.2014.00343

**Published:** 2014-12-01

**Authors:** Chong Chen, Qiuyang Zhang, Sen Liu, Mark Lambrechts, Yine Qu, Zongbing You

**Affiliations:** ^1^Department of Structural and Cellular Biology, Tulane Cancer Center and Louisiana Cancer Research Consortium, Tulane Center for Stem Cell Research and Regenerative Medicine, Tulane Center for Aging, Tulane University School of Medicine, New Orleans, LA, USA; ^2^Department of Orthopaedic Surgery, Tulane Cancer Center and Louisiana Cancer Research Consortium, Tulane Center for Stem Cell Research and Regenerative Medicine, Tulane Center for Aging, Tulane University School of Medicine, New Orleans, LA, USA; ^3^Department of Histology and Embryology, Hebei United University School of Basic Medicine, Tangshan, Hebei Province, China

**Keywords:** IL-17, insulin, IGF1, inflammation, prostate cancer, obesity

## Abstract

In the United States, one-third of population is affected by obesity and almost 29 million people are suffering from type 2 diabetes. Obese people have elevated serum levels of insulin, insulin-like growth factor 1 (IGF1), and interleukin-17 (IL-17). Insulin and IGF1 are known to enhance IL-17-induced expression of inflammatory cytokines and chemokines, which may contribute to the chronic inflammatory status observed in obese people. We have previously demonstrated that insulin/IGF1 signaling pathway crosstalks with IL-17-activated nuclear factor-κB pathway through inhibiting glycogen synthase kinase 3β (GSK3β) activity. However, it is unclear whether GSK3α also plays a role and whether this crosstalk can be manipulated by AZD5363, a novel pan-Akt inhibitor that has been shown to increase glycogen synthase kinase 3 activity through reducing phosphorylation of GSK3α and GSK3β. In this study, we investigated IL-17-induced expression of C-X-C motif ligand 1 (*Cxcl1*), C-C motif ligand 20 (*Ccl20*), and interleukin-6 (*Il-6*) in wild-type, GSK3α^−/−^, and GSK3β^−/−^ mouse embryonic fibroblast cells as well as in mouse prostate tissues by real-time quantitative PCR. We examined the proteins involved in the signaling pathways by Western blot analysis. We found that insulin and IGF1 enhanced IL-17-induced expression of *Cxcl1*, *Ccl20*, and *Il-6*, which was associated with increased phosphorylation of GSK3α and GSK3β in the presence of insulin and IGF1. AZD5363 inhibited the synergy between IL-17 and insulin/IGF1 through reducing phosphorylation of GSK3α and GSK3β by inhibiting Akt function. These findings imply that the cooperative crosstalk of IL-17 and insulin/IGF1 in initiating inflammatory responses may be alleviated by AZD5363.

## Introduction

Interleukin-17 (IL-17 or IL-17A) is an inflammatory cytokine (1). It can activate nuclear factor-κB (NF-κB) activator 1 (Act1) through similar expression to fibroblast growth factor genes, IL-17 receptors, and Toll–IL-1R (SEFIR) domains, upon its binding to a heterodimer of IL-17RA/IL-17RC receptor complex (2–6). Act1, as an E3 ubiquitin ligase, activates tumor necrosis factor receptor-associated factor 6 (TRAF6) through lysine-63-linked ubiquitination (7). The polyubiquitinated TRAF6 triggers transforming growth factor-β-activated kinase 1 (TAK1) and subsequently IκB kinase (IKK) complex, which in turn leads to activation of NF-κB pathway that induces transcription of a variety of cytokines, chemokines, and growth factor, e.g., C-X-C motif ligand 1 (Cxcl1) and IL-6 (8–10). Several studies have demonstrated that IL-17 stabilizes downstream *Cxcl1* mRNA through an inducible kinase IKKi-dependent Act1–TRAF2–TRAF5 complex, which ligands with splicing factor 2 [SF2, also named alternative splicing factor (ASF)] and prevents SF2/ASF-mediated mRNA degradation (11, 12).

Insulin is a hormone produced by the pancreas β cells, and its abnormal high concentration (hyperinsulinemia) may circulate in the body of people with obesity and type 2 diabetes mellitus with insulin resistance. Under hyperinsulinemic conditions, the liver produces insulin-like growth factor 1 (IGF1) (13). Two types of insulin receptors (IR-A and IR-B) can bind to either insulin or IGF1. IGF1 can also bind to a heterodimer of IR and IGF1 receptor (IGF1R). Upon binding with the receptors, insulin (or IGF1) leads to autophosphorylation of the β subunit of IR or IGF1R (14), which in turn recruits insulin receptor substrates-1 (IRS-1) to IRS4, and then phosphatidylinositol 3-kinase (PI3K)/Akt pathway is activated (8). One of the major substrates of Akt is glycogen synthase kinase 3β (GSK3β) (8, 15). Previous studies have shown that insulin inactivates GSK3β by inducing phosphorylation at serine 9 mainly via Akt signaling pathway (15, 16).

Glycogen synthase kinase 3 includes two type of isoforms GSK3α and GSK3β, which are ubiquitously expressed in all cells and capable of phosphorylating more than 50 substrates (17). One of the substrates, CAAT enhancer binding protein β (C/EBPβ), is also induced by IL-17 (3, 9, 18). C/EBPβ transcription factor is essential for transcription of IL-17 downstream target genes such as IL-6 and 24p3/lipocalin 2 (19). Phosphorylation of C/EBPβ inhibits expression of IL-17 downstream target genes, thus GSK3β negatively regulates IL-17 signaling through phosphorylation of C/EBPβ (20). Indeed, inhibition of glycogen synthase kinase 3 (GSK3) activity by GSK3 inhibitor can enhance IL-17-induced expression of IL-6, 24p3/lipocalin 2, CXCL5, C-C motif ligand 2 (CCL2), CCL7, and NF-κB inhibitor zeta, whereas, overexpression of GSK3β can inhibit IL-17-induced IL-6 promoter and 24p3 promoter activities in a mouse stromal ST2 cell line (21). Therefore, GSK3β functions as an intrinsic negative regulator of IL-17-mediated inflammatory responses (21). Our previous study has shown that GSK3β inhibition by phosphorylation or gene knockout enhanced IL-17-induced expression of inflammatory cytokines and chemokines (8).

AZD5363 [(*S*)-4-amino-*N*-[1-(4-chlorophenyl)-3-hydroxypropyl]-1-(7*H*-pyrrolo [2, 3-d] pyrimidin-4-yl) piperidine-4-carboxamide] is a pan-Akt inhibitor that is currently being investigated in phase I clinical trials for cancer therapy (22, 23). Akt is a serine/threonine protein kinase, also known as protein kinase B (PKB), which regulates a variety of cellular process including cell proliferation, cell survival, and glucose and fatty acid metabolism (24–26). Because Akt signaling network is the key pro-tumor network in human cancers, it is a target in development of new therapies (27). The active form of Akt is phosphorylated Akt (P-Akt), which may occur at threonine 308 (Thr308) residue phosphorylated by 3-phosphoinositide dependent protein kinase 1 (PDK1), or at serine 473 (Ser 473) residue phosphorylated by mTor complex 2 (mTORC2) (28–30). Given that GSK3 is a downstream substrate of Akt, we hypothesized that inhibition of Akt by AZD5363 might inhibit the synergistic effects between IL-17 and insulin/IGF1. In this study, we tested this hypothesis.

## Materials and Methods

### Cells and tissue culture

Mouse embryonic fibroblast cells (wild-type, GSK3α^−/−^, or GSk3β^−/−^ gene knockout) (31) were maintained in a 37°C, 5% CO_2_ humidified incubator. All of these cell lines express IL-17 receptors A and C (data not shown). Dulbecco’s Modified Eagle’s Medium (DMEM; Mediatech, Inc., Manassas, VA, USA) with 10% fetal bovine serum (FBS; Mediatech, Inc.) and 1% penicillin/streptomycin was used as the growth medium. Mouse prostate tissues were dissected from 7 to 9-week-old male mice euthanized by CO_2_ asphyxiation. The prostate tissues were washed three times with phosphate-buffered saline (PBS), cut into 1–2 mm^3^ cubes, and kept in 60-mm cell culture dishes in serum-free DMEM in the incubator. The animal study was approved by the Animal Care and Use Committee of Tulane University.

### Treatment of cells and tissues

Mouse embryonic fibroblast cells were seeded into 60-mm cell culture dishes with 0.5 × 10^6^ cells/dish. After 24 h incubation, the cells were incubated with serum-free DMEM for 20 h, and then treated with IL-17 (R&D Systems, Inc., Minneapolis, MN, USA), insulin, IGF1 (Sigma Aldrich, Inc., St Louis, MO, USA), and/or AZD5363 (Selleck Chemicals, Inc., Houston, TX, USA). The harvested mouse prostate tissues immersed in serum-free DMEM were incubated for 20 h before any treatments. The treatment for cells and tissues included: (1) control with vehicle; (2) AZD5363 at 2 μM for 3 h; (3) insulin at 50 ng/ml for 2.5 h; (4) IGF1 at 50 ng/ml for 2.5 h; (5) IL-17 at 20 ng/ml for 2 h; (6) insulin + IL-17 at the same doses but adding insulin 0.5 h before addition of IL-17; (7) IGF1 + IL-17 at the same doses but adding IGF1 0.5 h before addition of IL-17; (8) AZD5363 + Insulin + IL-17 at the same doses but adding AZD5363 1 h and insulin 0.5 h before addition of IL-17; and (9) AZD5363 + IGF1 + IL-17 at the same doses but adding AZD5363 1 h and IGF1 0.5 h before addition of IL-17.

### Real-time quantitative reverse transcriptase PCR

Following treatments, mouse embryonic fibroblast (MEF) cells or mouse prostate tissues were collected in lysis buffer. Mouse prostate tissues were homogenized with Fisher Scientific™ Model 505 sonic dismembrator. Total RNAs of MEF cells or mouse prostate tissues were isolated by using RNeasy Kit (QIAGEN, Valencia, CA, USA) according to the manufacturer’s instructions. Genomic DNA contamination of each sample was avoided by using DNase I digestion. RNA was reversed to cDNA by using iScript™ cDNA synthesis kit (Bio-rad Laboratories, Hercules, CA, USA). Mouse glyceraldehyde-3-phosphate dehydrogenase (*Gapdh*), *Cxcl1*, *Ccl20*, and *Il-6* primers were obtained from Eurofins (Huntsville, AL, USA). The PCR primers specific for each gene were as follows: *Cxcl1* forward: 5′-CACCCAAACCGAAGTCATAG-3′, reverse: 5′-AAGCCAGCGTTCACCAGA-3′; *Ccl20* forward: 5′-AACTGGGTGAAAAGGGCTGT-3′, reverse: 5′-GTCCAATTCCATCCCAAAAA-3′; *Il-6* forward: 5′-CTACCCCAATTTCCAATGCT-3′, reverse: 5′-ACCACAGTGAGGAATGTCCA-3′; *Gapdh* forward: 5′-TGCACCACCAACTGCTTAG-3′, reverse: 5′-GGATGCAGGGATGATGTTC-3′. Quantitative real-time PCR (qRT-PCR) was conducted using iQ5^®^ iCycler and iQ™ SYBR Green Supermix (Bio-Rad Laboratories) following the manufacturer’s protocols. The result of each group was normalized to its own *Gapdh* level by using the formula ΔCt (Cycle threshold) = Ct of target gene − Ct of *Gapdh*. The fold change of mRNA level of each treatment group was calculated as: ΔΔCt = ΔCt of target gene in the treatment group − ΔCt of target gene in control group, and fold change = 2^−ΔΔCt^.

### Western blot analysis

Following the treatment of cells or tissues, proteins were extracted by using RIPA lysis buffer, which contains 50 mM sodium fluoride, 0.5% Igepal CA-630 (NP-40), 10 mM sodium phosphate, 150 mM sodium chloride, 25 mM Tris (pH 8.0), 1 mM phenylmethylsulfonyl fluoride, 2 mM ethylenediaminetetraacetic acid (EDTA), and 1.2 mM sodium vanadate. Protein concentration was assessed by using Bio-Rad Protein Assay Dye Reagent Concentrate (Bio-Rad Laboratories, Hercules, CA, USA) and BioTek ELx800 microplate reader (BioTek, Winooski, VT, USA). Eighty microgram of protein of each group was loaded to 10% SDS-polyacrylamide gel electrophoresis and transferred to polyvinylidene difluoride membrane. Membrane blocking was done using 5% non-fat dry milk in TBST buffer (25 mM Tris-HCl, 125 mM sodium chloride, and 0.1% Tween 20). Primary antibody was incubated with the membrane at 4°C overnight. The membrane was washed three times with TBST, and incubated with IRDye^®^ 800CW- or IRDye^®^ 680RD-conjugated secondary antibodies (LI-COR Biosciences, Lincoln, NE, USA) at room temperature for 1 h. The membrane was scanned by Odyssey Infrared Imager (LI-COR Biosciences) for visualization. The antibodies used included: rabbit anti-P-Akt (S473), rabbit anti-Akt, rabbit anti-P-GSK3α (S21), rabbit anti-GSK3α, rabbit anti-P-GSK3β (S9), and rabbit anti-GSK3β antibodies were purchased from Cell Signaling Technology, Danvers, MA, USA. Mouse anti-GAPDH antibody was purchased from Millipore, Billerica, MA, USA.

### Statistical analysis

The data were presented as mean ± SD of triplicate experiments (*n* = 3). Statistical significance was determined by one-way ANOVA and Tukey’s tests. All of the analyses were performed using GraphPad Prism^®^ 5.0 (GraphPad Software, La Jolla, CA, USA).

## Results

**Figure 1:**
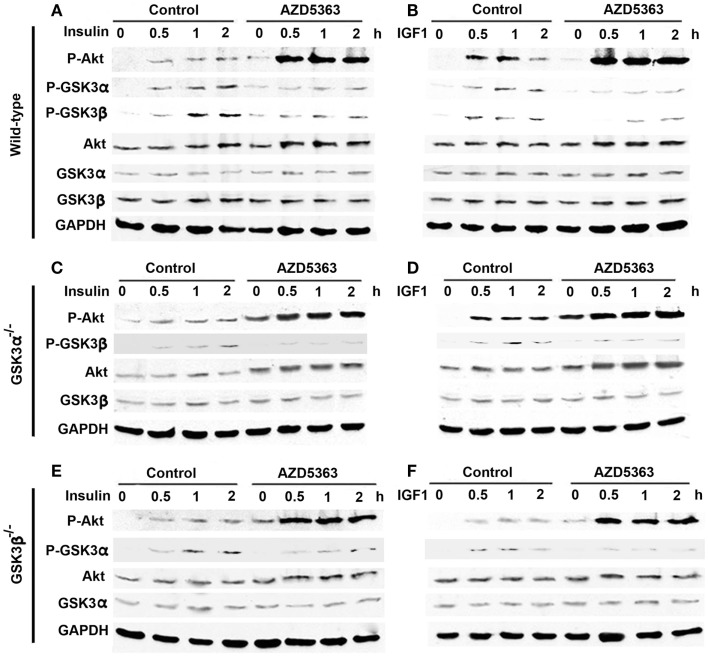
**Effects of AZD5363 on insulin/IGF1 signaling pathways**. **(A)** Effects of insulin with or without AZD5363 on wild-type MEF cells; **(B)** Effects of IGF1 with or without AZD5363 on wild-type MEF cells; **(C)** Effects of insulin with or without AZD5363 on GSK3α^−/−^ MEF cells; **(D)** Effects of IGF1 with or without AZD5363 on GSK3α^−/−^ MEF cells; **(E)** Effects of insulin with or without AZD5363 on GSK3β^−/−^ MEF cells; **(F)** Effects of IGF1 with or without AZD5363 on GSK3β^−/−^ MEF cells. The concentrations of insulin and IGF1 were 50 ng/ml and the concentration of AZD5363 was 2 μM. The levels of phosphorylated and unphosphorylated Akt, GSK3α, and GSK3β were shown by western blot analysis. Equal loading of proteins was confirmed by reprobing GAPDH.

In the wild-type MEF cells, insulin or IGF1 alone treatment led to increased levels of P-Akt, P-GSK3α, and P-GSK3β (Figures 1A,B). When AZD5363 treatment was added, the levels of P-Akt were further increased. On the contrary, AZD5363 treatment reduced the levels of P-GSK3α and P-GSK3β (Figures 1A,B). In the GSK3α^−/−^ MEF cells (Figures 1C,D) and GSK3β^−/−^ MEF cells (Figures 1E,F), insulin or IGF1 alone treatment increased the levels of P-Akt, and subsequently the levels of P-GSK3β and P-GSK3α in GSK3α^−/−^ and GSK3β^−/−^ MEF cells, respectively. AZD5363 treatment led to a further increase of P-Akt compared to insulin or IGF1 alone treatment in both GSK3α^−/−^ and GSK3β^−/−^ MEF cells. However, AZD5363 treatment reduced the levels of P-GSK3β and P-GSK3α in GSK3α^−/−^ and GSK3β^−/−^ MEF cells, respectively, in comparison to insulin or IGF1 alone treatment.

**Figure 2:**
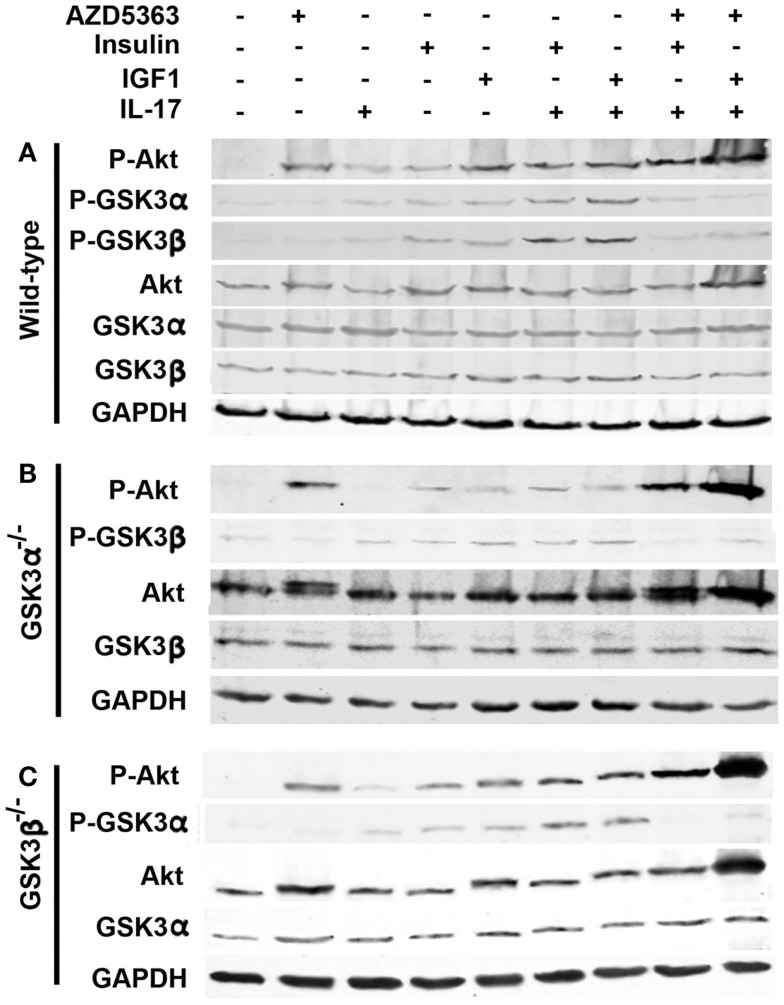
**Effects of AZD5363 on IL-17 and insulin/IGF1 signaling pathways in wild-type MEF cells (A), GSK3α^−/−^ MEF cells (B), and GSK3β^−/−^ MEF cells (C)**. Cells were treated with 20 ng/ml IL-17, 50 ng/ml insulin, 50 ng/ml IGF1, and 2 μM AZD5363, either alone or in combination for 2 h. The levels of phosphorylated and unphosphorylated Akt, GSK3α, and GSK3β were shown by western blot analysis. Equal loading of proteins was confirmed by reprobing GAPDH.

As shown in Figure 2A, IL-17, insulin or IGF1 alone treatment only slightly increased the levels of P-Akt, P-GSK3α, and P-GSK3β in wild-type MEF cells, compared to control group. A combination of insulin and IL-17, or IGF1 and IL-17, further increased the levels of P-Akt, P-GSK3α, and P-GSK3β. When AZD5363 treatment was added to the combined treatment groups, the levels of P-GSK3α and P-GSK3β were dramatically reduced, though the levels of P-Akt were further increased. In GSK3α^−/−^ and GSK3β^−/−^ MEF cells, similar changes were observed, except that only GSK3β (Figure 2B) or GSK3α (Figure 2C) was present due to knockout of the other GSK3 isoform.

**Figure 3:**
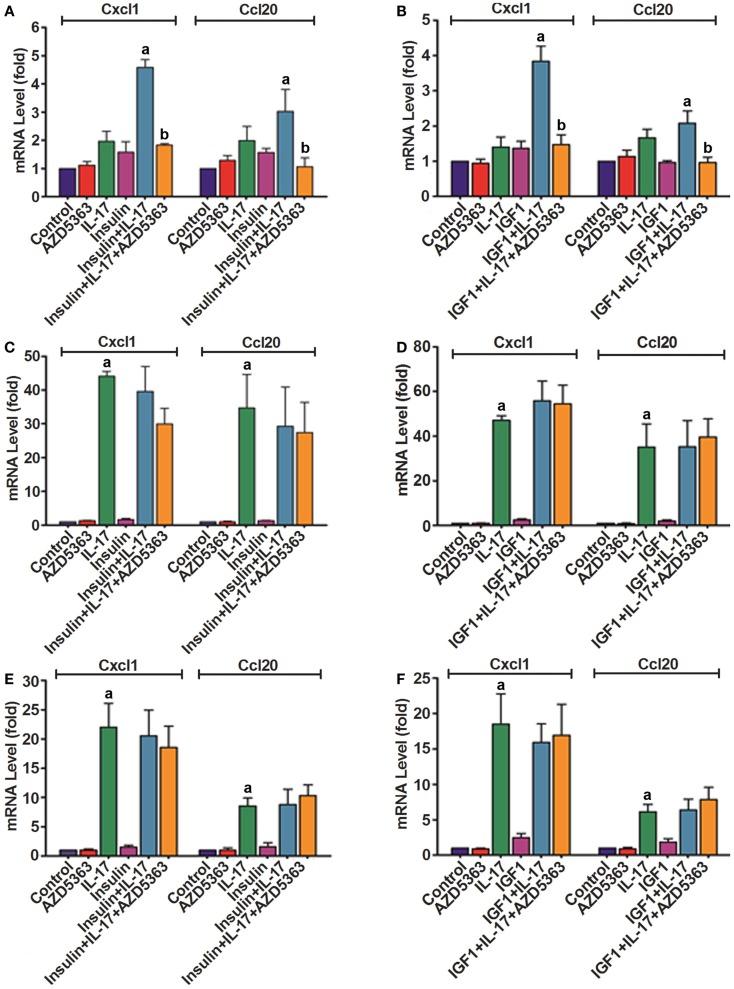
**Expression of *Cxcl1* and *Ccl20* mRNAs in wild-type MEF cells (A,B), GSK3α^−/−^ MEF cells (C,D), and GSK3β^−/−^ MEF cells (E,F)**. Cells were treated with 20 ng/ml IL-17, 50 ng/ml insulin, 50 ng/ml IGF1, and 2 μM AZD5363, either alone or in combination for 2 h. The levels of *Cxcl1* and *Ccl20* mRNAs were determined using real-time PCR. Data represent mean ± SD of triplicate experiments (*n* = 3). a, *p* < 0.05 Compared to IL-17 alone or insulin/IGF1 alone; b, *p* < 0.05 compared to the combination of IL-17 and insulin or IGF1.

Because AZD5363 treatment decreased the levels of P-GSK3α and P-GSK3β that might affect IL-17-induced gene expression (8), we checked the mRNA levels of *Cxcl1* and *Ccl20* in wild-type, GSK3α^−/−^ and GSK3β^−/−^ MEF cells after the treatment as described above. In the wild-type MEF cells, IL-17 or insulin alone treatment increased *Cxcl1* mRNA levels by 2.0 ± 0.4 or 1.6 ± 0.8-fold, compared to control group (Figure 3A). *Cxcl1* mRNA level was increased by 4.6 ± 0.6-fold in the insulin and IL-17 combined treatment group, which was statistically significant compared to insulin or IL-17 alone treatment group (*p* < 0.05). Addition of AZD5363 to this combined treatment group reduced *Cxcl1* mRNA level to 1.8 ± 0.1-fold, which was significantly less than the insulin and IL-17 combined treatment group (Figure 3A, *p* < 0.05). Similarly, *Ccl20* mRNA levels were increased by 2.0 ± 0.5 and 1.6 ± 0.3-fold in IL-17 or insulin alone treated group, respectively. A combination of insulin and IL-17 treatment increased *Ccl20* mRNA level by 3.0 ± 0.8-fold, which was significantly higher than either IL-17 or insulin alone treatment. In contrast, addition of AZD5363 to the combined treatment reduced *Ccl20* mRNA level almost to the basal level of 1.1 ± 0.3-fold, which was significantly lower than the insulin and IL-17 combined treatment group (Figure 3A, *p* < 0.05). As shown in Figure 3B, IGF1 and IL-17 also synergistically induced *Cxcl1* and *Ccl20* mRNA expression, which was inhibited by addition of AZD5363. In GSK3α^−/−^ (Figures 3C,D) and GSK3β^−/−^ (Figures 3E,F) MEF cells, IL-17 alone treatment dramatically increased the levels of *Cxcl1* and *Ccl20* mRNA. In contrast to wild-type MEF cells, combination of insulin or IGF1 with IL-17 did not further increase levels of *Cxcl1* and *Ccl20* mRNA, compared to IL-17 alone treatment (Figures 3C–F). Furthermore, addition of AZD5363 to the combined treatment did not reduce the elevated mRNA levels of *Cxcl1* or *Ccl20* (Figures 3C–F).

**Figure 4:**
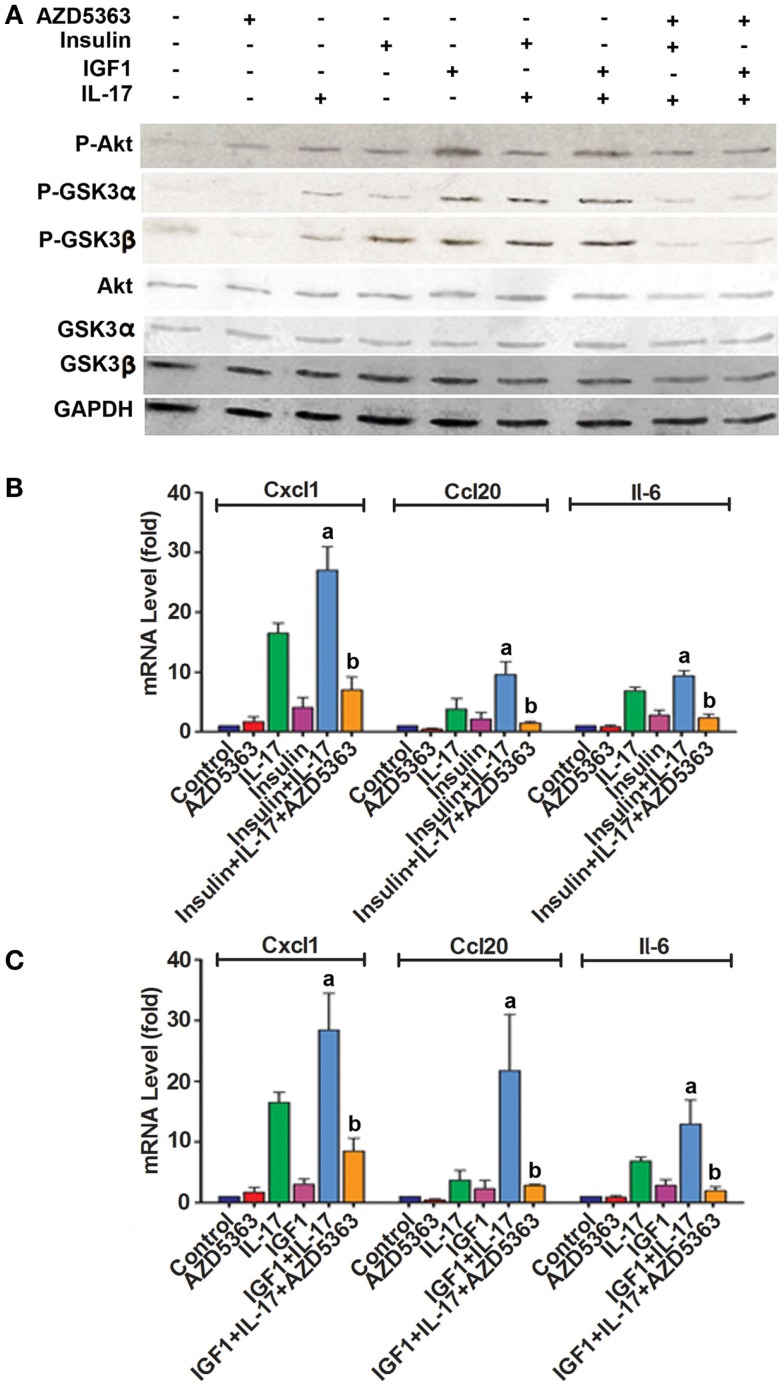
**Effects of AZD5363 on IL-17 and insulin/IGF1 signaling pathways and expression of *Cxcl1* and *Ccl20* mRNAs in mouse prostate tissues**. Mouse prostate tissues were cultured *ex vivo* and treated with 20 ng/ml IL-17, 50 ng/ml insulin, 50 ng/ml IGF1, and 2 μM AZD5363, either alone or in combination for 2 h. **(A)** The levels of phosphorylated and unphosphorylated Akt, GSK3α and GSK3β were shown by western blot analysis. Equal loading of proteins was confirmed by reprobing GAPDH. **(B,C)** The levels of *Cxcl1* and *Ccl20* mRNAs were determined using real-time PCR. Data represent mean ± SD of triplicate experiments (*n* = 3). a, *p* < 0.05 Compared to IL-17 alone or insulin/IGF1 alone; b, *p* < 0.05 compared to the combination of IL-17 and insulin or IGF1.

In order to assess if our findings in the studies of cell lines are relevant to the *in vivo* organ tissues, we did similar experiments using *ex vivo* cultured mouse prostate tissues. As shown in Figure 4A, increased levels of P-Akt, P-GSK3α, and P-GSK3β were observed in mouse prostate tissues treated with insulin alone, IGF1 alone, a combination of insulin and IL-17, and a combination of IGF1 and IL-17, compared to the control group. However, addition of AZD5363 to the combined treatment groups reduced the levels of P-GSK3α and P-GSK3β, compared to the combined treatment groups. The changes in the signaling proteins were associated with the changes in the mRNA levels of *Cxcl1*, *Ccl20*, and *Il-6*. As shown in Figure 4B, a combination of insulin and IL-17 treatment significantly increased the mRNA levels of *Cxcl1*, *Ccl20*, and *Il-6*, compared to insulin or IL-17 alone treatment (*p* < 0.05). Similarly, a combination of IGF1 and IL-17 treatment showed the same effects (Figure 4C). However, when AZD5363 was added to the combined treatment groups, the induction of mRNA levels of *Cxcl1*, *Ccl20*, and *Il-6* was significantly reduced, compared to the combined treatment groups without AZD5363 (Figures 4B,C).

## Discussion

**Figure 5:**
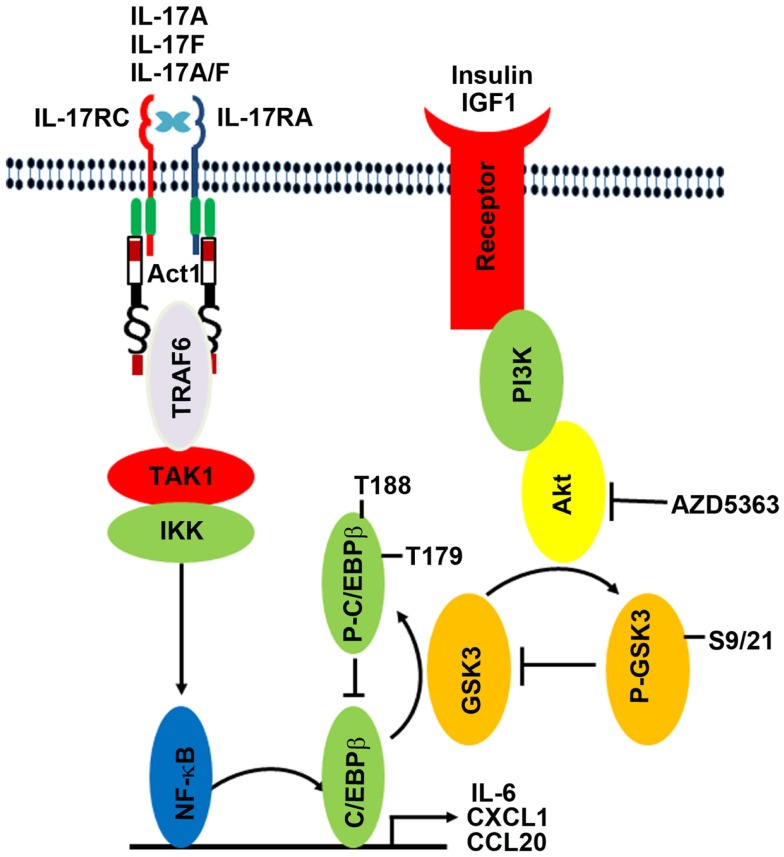
**Illustration of the proposed crosstalk between insulin/IGF1 and IL-17 signaling pathways**. IL-17 acts through the IL-17RA:IL-17RC receptor complex to activate Act1–TRAF6–TAK1–IKK signaling cascade, thus activating NF-κB transcription factor and subsequently activating C/EBPβ transcription factors. NF-κB and C/EBPβ transcription factors are required for initiation of transcription of the downstream target genes such as *IL-6*, *Cxcl1*, and *Ccl20*. Insulin and IGF1 bind to their receptors and activate PI3K/Akt pathway; Akt phosphorylates GSK3B at serine 9 and GSK3A at serine 21 to inhibit GSK3 activity; GSK3 phosphorylates C/EBPβ at threonine 179 after a priming phosphorylation at threonine 188 by ERK1/2, thus inhibiting C/EBPβ’s transcription function. Therefore, insulin/IGF1 signaling is linked with IL-17 signaling by GSK3 and C/EBPβ. AZD5363 inhibits Akt activation, thus enhancing GSK3 activity and subsequently diminishing IL-17-induced gene expression by inhibiting C/EBPβ function.

Inflammation has been shown to be a driving force behind a variety of cancer types (32–34). IL-17 is an inflammatory cytokine that stimulates leukocytes, fibroblasts, epithelial cells, and endothelial cells to release inflammatory signals that can further fire up inflammation (1). We have previously demonstrated that IL-17 promotes formation and growth of prostate cancer in a mouse model (35, 36). Recently, we showed that insulin and IGF1 enhance IL-17-induced expression of inflammatory cytokines and chemokines (8). The crosstalk between insulin/IGF1 signaling pathway and IL-17 signaling pathway is mediated by GSK3β, as GSK3β knockout blocks the crosstalk. In the present study, we found that GSK3α knockout also blocks the crosstalk between insulin/IGF1 and IL-17 pathways. In fact, knockout of either GSK3α or GSK3β appears to relieve the repressive function of GSK3 on IL-17-induced gene expression, as IL-17 can induce gene expression to the levels significantly higher than in the wild-type MEFs where IL-17 can usually induce gene expression to very modest levels. These findings suggest that both GSK3α and GSK3β isoforms are required to be present, in order to repress IL-17-induced gene expression. Lithium chloride is an inhibitor to both GSK3α and GSK3β isoforms, which has been shown to increase IL-17-induced gene expression in two previous studies (8, 20). The exact molecular mechanisms underlying the crosstalk are yet to be determined, though a previous study suggested that it might be phosphorylation of C/EBPβ by GSK3, which inhibits the transcription function of C/EBPβ (21). As shown in Figure 5, IL-17 acts through the IL-17RA:IL-17RC receptor complex to activate Act1–TRAF6–TAK1–IKK signaling cascade, thus activating NF-κB transcription factor and subsequently activating C/EBPβ transcription factors. NF-κB and C/EBPβ transcription factors are required for initiation of transcription of the downstream target genes such as *IL-6*, *Cxcl1*, and *Ccl20*. Insulin and IGF1 bind to their receptors and activate PI3K/Akt pathway; Akt phosphorylates GSK3B at serine 9 and GSK3A at serine 21 to inhibit GSK3 activity; GSK3 phosphorylates C/EBPβ at threonine 179 after a priming phosphorylation at threonine 188 by ERK1/2, thus inhibiting C/EBPβ’s transcription function. Therefore, insulin/IGF1 signaling is linked with IL-17 signaling by GSK3 and C/EBPβ. AZD5363 inhibits Akt activation, thus enhancing GSK3 activity and subsequently diminishing IL-17-induced gene expression by inhibiting C/EBPβ function.

Manipulation of the crosstalk between insulin/IGF1 and IL-17 is potentially significant in obese population. It has been reported that serum and tissue levels of IL-17 are increased in obese mice (37, 38) and humans (39). Interestingly, serum levels of insulin and IGF1 are also increased in obese population, which together with IL-17, may be the underlying cause of the chronic inflammatory state with increased serum levels of inflammatory mediators TNFα and IL-6 (8, 40). Obesity has been associated with increased risks of breast cancer, endometrial cancer, esophageal adenocarcinoma, pancreas cancer, colorectal cancer, renal cancer, thyroid cancer, gallbladder cancer, and prostate cancer (41–49). Chronic inflammation in obesity is suspected as one of the possible mechanisms underlying the increased cancer risk. In our previous study, we found that melatonin can block the crosstalk between insulin/IGF1 and IL-17 through inhibition of Akt function (8). In the present study, we found that AZD5363, a pan-Akt inhibitor, can do the same. AZD5363 reduced phosphorylation of GSK3α at serine 21 and GSK3β at serine 9, thus increasing the enzyme activities of GSK3α and GSK3β, and subsequently represses IL-17-induced gene expression. Preclinical studies have shown that AZD5363 may be effective in inhibiting tumor growth (27), yet it remains to be determined whether AZD5363 may alter the inflammatory microenvironment in the tumors and how this contributes to the anti-tumor function of AZD5363.

Interestingly, we observed that AZD5363, a pan-Akt inhibitor, increased the P-Akt levels in wild-type, GSK3α^−/−^ and GSK3β^−/−^ MEF cells. In general, phosphorylated Akt is the activated form of Akt (30). However, it has been reported that several Akt inhibitors elevate the levels of P-Akt. The mechanism behind this may be that suppression of S6K (p70S6K) activity stabilizes IRS-1 and increases IRS-1 adapter protein levels, which in turn induces Akt activity (50–54). Another possible cause of the hyperphosphorylation is that the Akt inhibitor sensitizes the pleckstrin homology (PH) domain to bind basal levels of PIP3 to facilitate membrane localization and induce conformational change of Akt to become more susceptible to kinase phosphorylation or less susceptible to phosphatase dephosphorylation (55). Of note, the increase of P-Akt and total Akt was less obvious in the mouse prostate tissues, compared to the MEFs upon AZD5363 treatment. We speculate that this might be due to that the prostate glandular tissues responded differently from the MEFs. But the exact reason is not clear.

In summary, this study indicates that insulin and IGF1 can enhance IL-17-induced inflammatory responses through suppression of GSK3 function by phosphorylation of GSK3α and GSK3β. AZD5363 inhibits Akt function and thus inhibits the synergy between IL-17 and insulin/IGF1 through enhancing GSK3 function by reducing phosphorylation of GSK3α and GSK3β. These findings imply that the cooperative crosstalk of IL-17 and insulin/IGF1 in initiating inflammatory responses may be alleviated by AZD5363.

## Author Contributions

Chong Chen performed the experiments, analyzed the data, and prepared the manuscript. Qiuyang Zhang, Mark Lambrechts, Sen Liu, and Yine Qu participated in the experiments and analysis of data. Zongbing You conceived and designed the work, analyzed the data, and prepared the manuscript. All authors critically revised the manuscript, approved the final version, and agreed to be accountable for all aspects of the manuscript.

## Conflict of Interest Statement

The authors declare that the research was conducted in the absence of any commercial or financial relationships that could be construed as a potential conflict of interest.

## Abbreviations

Act1, NF-κB activator 1; ASF, alternative splicing factor; CCL2, C-C motif ligand 2; Ccl20, C-C motif ligand 20; CCL7, C-C motif ligand 7; cDNA, complementary deoxyribonucleic acid; C/EBPβ, CAAT enhancer binding protein β; Cxcl1, C-X-C motif ligand 1; CXCL5, C-X-C motif ligand 5; DMEM, Dulbecco’s modified eagle’s medium; Gapdh, glyceraldehyde-3-phosphate dehydrogenase; GSK, glycogen synthase kinase; IGF1, insulin-like growth factor 1; IGF1R, insulin-like growth factor 1 receptor; IKK, IκB kinase; IL-6, interleukin-6; IL-17, interleukin-17; IL-17R, interleukin-17 receptor; IR, insulin receptor; IRS, insulin receptor substrates; MEF, mouse embryonic fibroblast; mTORC2, mTor complex 2; NF-κB, nuclear factor-κB; PDK1, protein kinase 1; PH, pleckstrin homology; PI3K, phosphatidylinositol 3-kinase; SF2, splicing factor 2; SEFIR, similar expression to fibroblast growth factor genes, IL-17 receptors, and Toll–IL-1R; TAK1, transforming growth factor-β-activated kinase 1; TRAF6, tumor necrosis factor receptor-associated factor 6.
